# The Role of Viral Load in COVID-19-Induced Encephalitis

**DOI:** 10.3390/jcm15051833

**Published:** 2026-02-27

**Authors:** Lerzan Dogan, Dilaver Kaya, Neval Yurtturan Uyar, Alp Dincer, Sesin Kocagoz, Bulent Gucyetmez, Ibrahim Ozkan Akinci

**Affiliations:** 1 Department of Anesthesiology and Reanimation, Acibadem Mehmet Ali Aydınlar University, Istanbul 34752, Turkey; 2 Department of Neurology, Acibadem Mehmet Ali Aydınlar University School of Medicine, Istanbul 34752, Turkey; 3 Department of Medical Microbiology, Acibadem Mehmet Ali Aydınlar University School of Medicine, Istanbul 34752, Turkey; 4 Department of Radiology, Acibadem Mehmet Ali Aydınlar University School of Medicine, Istanbul 34752, Turkey; 5 Department of Infectious Diseases and Clinical Microbiology, Acibadem Mehmet Ali Aydınlar University School of Medicine, Istanbul 34752, Turkey; sesin.kocagoz@acibadem.com; 6 Intensive Care Unit, Acibadem Healthcare Group, Istanbul 34750, Turkey

**Keywords:** COVID-19, encephalitis, neurological complications, SARS-CoV-2, viral load

## Abstract

**Introduction:** Acute encephalitis is a severe neurological complication whose association with SARS-CoV-2 infection is increasingly recognized. However, the precise pathophysiological mechanisms remain incompletely understood. Understanding the factors contributing to central nervous system involvement in COVID-19 is crucial for guiding clinical management and improving patient outcomes. **Methods:** This single-center, retrospective cohort study analyzed data from 450 adult critically ill patients with RT-qPCR-confirmed SARS-CoV-2 infection admitted to our ICU between May 2021 and March 2023. All SARS-CoV-2-positive patients with suspected CNS involvement were included and categorized into encephalitis-positive (E+, *n* = 38) and encephalitis-negative (E−, *n* = 58) groups according to neurological examination, imaging, and lumbar puncture findings during ventilator weaning. Key patient characteristics, laboratory parameters at ICU admission (including SARS-CoV-2 Ct values and D-dimer levels), and clinical outcomes were analyzed with appropriate statistical methods, including ROC curve analysis and Cox regression. **Results:** Patients in the E+ group, compared with the E− group, were significantly older (mean 69 ± 15 vs. 61 ± 12 years, *p* = 0.006) and exhibited significantly lower median SARS-CoV-2 Ct values (23.7 vs. 27.0, *p* < 0.001) indicative of higher viral loads. The median D-dimer levels were also significantly elevated in the E+ group (4.6 vs. 1.1, *p* < 0.001). Other baseline characteristics and inflammatory markers were comparable between groups. Patients with encephalitis experienced significantly longer mechanical ventilation durations (median 19 vs. 14 days, *p* = 0.006) and ICU stays (median 21 vs. 15 days, *p* = 0.009) compared to those without encephalitis. No significant difference was observed in overall mortality between the groups (50.0% vs. 56.9%, *p* = 0.507). Multivariate analysis identified lower Ct values (HR: 1.9, *p* = 0.032) and higher D-dimer levels (HR: 2.9, *p* < 0.010) at ICU admission as independent risk factors for encephalitis development. **Conclusions:** Our findings indicated that higher SARS-CoV-2 viral loads (lower Ct values), older age, and higher D-dimer levels were significantly associated with a greater risk of COVID-19-associated encephalitis in critically ill patients. These markers might aid in identifying patients at high risk of neurological complications, thereby facilitating earlier monitoring and potentially improving patient management. Further prospective studies are warranted to fully elucidate the pathophysiological mechanisms underlying this association.

## 1. Introduction

The COVID-19 pandemic has highlighted a significant link between SARS-CoV-2 infection and the development of encephalitis [1], presenting with diverse clinical manifestations and varied pathophysiological underpinnings [2,3,4]. Neurological complications in COVID-19 patients are frequently associated with an adverse prognosis [5]. Despite considerable research, a comprehensive understanding of COVID-19-associated encephalitis pathogenesis remains incomplete.

Recent evidence indicates that SARS-CoV-2 can trigger autoimmune responses [6], potentially contributing to severe outcomes such as intensive care unit (ICU) admission and increased mortality in susceptible individuals. However, the precise relationship between viral load and the induction of autoimmunity, as well as the broader pathophysiological mechanisms driving encephalitis and CNS involvement by SARS-CoV-2, is still unclear.

A deeper understanding of the interplay between viral load, disease severity, and viral transmissibility is fundamental for elucidating COVID-19 pathogenesis and developing more effective therapeutic and preventive strategies [7]. Despite this imperative, data specifically investigating the factors contributing to SARS-CoV-2 CNS involvement, its response to treatment, and subsequent patient outcomes remain limited [2]. While it is well-established that SARS-CoV-2 viral load, alongside patient age and comorbidities, can predict individuals at high risk for severe COVID-19 [8,9,10], its specific utility in identifying those prone to CNS complications is yet to be determined. Clarifying this relationship is critical, as it has direct implications for guiding treatment decisions and optimizing patient outcomes.

## 2. Methods and Design

This single-center, retrospective cohort study was conducted at a large tertiary ICU. Data were collected from patient records from 1 May 2021 to March 2023. Ethical approval was obtained (number 2024-4/154), and the requirement for written informed consent was waived because of the retrospective nature of the study. Our study adhered to the Strengthening the Reporting of Observational Studies in Epidemiology (STROBE) checklist guidelines.

The study cohort comprised adult patients (>18 years of age) admitted to the ICU due to acute respiratory failure secondary to confirmed SARS-CoV-2 infection. All SARS-CoV-2-positive patients presenting with suspected CNS involvement were included in the analysis. Key patient characteristics, ICU severity scores such as APACHE II (Acute Physiology and Chronic Health Evaluation II) and SOFA (Sequential Organ Failure Assessment), laboratory results at ICU admission (including leukocyte, C-reactive protein [CRP], procalcitonin, leukocyte/lymphocyte counts, ferritin, and lactate dehydrogenase [LDH]), viral load data, and clinical outcomes were systematically collected. Patients’ clinical courses were followed and recorded throughout their hospitalization.

### 2.1. SARS-CoV-2 Viral Load Assessment

Nasopharyngeal specimens were collected from all individuals upon ICU admission and subjected to quantitative Reverse Transcription Quantitative Polymerase Chain Reaction (RT-qPCR) assays for SARS-CoV-2. RT-qPCR serves as the gold standard for quantitative viral Ribonucleic Acid (RNA) assessment. The Cycle Threshold (Ct) value—the number of amplification cycles required for the fluorescence signal to cross a defined threshold—is inversely correlated with the initial viral load present in the sample. Lower Ct values indicate higher viral nucleic acid concentrations [11].

RNA isolation was performed manually using a viral nucleic acid isolation kit (vNAT^®^; Bioeksen, Istanbul, Turkey). Reverse transcription and RT-qPCR were performed on a Rotor-Gene Q (QIAGEN, Hilden, Germany) device with a Biospeedy^®^ kit (Bioeksen, Istanbul, Turkey). The ORF1ab and N gene regions of SARS-CoV-2 RNA were targeted, whereas the human ribonuclease P gene served as an internal control for sample quality and assay functionality. The BioSpeedy^®^ kit targets both ORF1ab and N genes; the ORF1ab gene Ct values were used for the standardized reporting and statistical analysis in this study. COVID-19 positivity was determined by the detection of the amplification curves of the RdRp gene region (8).

### 2.2. Diagnosis of COVID-19-Associated Encephalopathy/Encephalitis with Inflammatory Features

Given the high frequency of confounding factors in the ICU setting (e.g., metabolic derangements, sedation effects, ICU delirium), our diagnostic protocol was designed to minimize these influences. There were no significant differences in the primary treatment protocols between the groups. Crucially, neurological investigation (MRI and Lumbar Puncture [LP]) was deferred until the ventilator weaning period, following the resolution of acute metabolic derangements and the discontinuation of deep sedation. We acknowledge that delaying formal neurological evaluation until the ventilator weaning phase, while a pragmatic compromise necessary for patient safety and feasibility in the critical care setting, likely leads to an underestimation of the true incidence of COVID-19-associated CNS complications, particularly hyperacute syndromes. While earlier, less formalized bedside neurological assessments were performed during peak illness, these were not systematically documented or deemed suitable for definitive case classification. Our cohort, therefore, focuses primarily on patients exhibiting persistent or subacute/delayed CNS complications during the recovery phase. A neurological examination by a consultant was conducted when prolonged altered mental status (unconsciousness) extended beyond 48 h after discontinuation of deep sedation or when agitated delirium was refractory to standard management. Neurological investigation (cranial MRI and lumbar puncture) was initiated only in these selected patients exhibiting the persistent or refractory symptoms described above. Cases exhibiting clear signs of awakening were excluded from further neurological investigation.

### 2.3. Diagnostic Criteria and Terminology

Due to the consistently negative SARS-CoV-2 CSF PCR results observed in this cohort, the clinical syndrome is unlikely to represent direct viral encephalitis, but rather a para- or post-infectious, immune-mediated, or vascular pathology. Accordingly, the condition is henceforth referred to as COVID-19-associated encephalitis/encephalopathy throughout the manuscript to reflect this likely indirect etiology and avoid implying direct viral induction.

The diagnosis was established during the ventilator weaning period, based on the presence of:

1. Altered mental status or seizures,
2. At least one objective finding from comprehensive infectious screening (excluding common non-SARS-CoV-2 causes), CSF analysis, or characteristic findings on cranial MRI.

Cerebrospinal Fluid Analysis: Analysis from LP included measurement of protein, glucose, IgG levels, and cell count; viral PCR (for both COVID-19 and common seasonal viruses); and oligoclonal band testing. Patients were subsequently categorized into two groups: those diagnosed with encephalitis (E+ group) and those without encephalitis (E− group).

### 2.4. Statistical Analysis

SPSS version 29 was used for statistical analyses (IBM Corp., Armonk, NY, USA). The data are presented as means with standard deviations, medians with quartiles, and percentages. Normal distribution was assessed with the Kolmogorov–Smirnov test, Student’s *t*-test, or Mann–Whitney U test to compare continuous variables between E+ and E− groups, or with the chi-square test or Fisher’s exact test to compare categorical variables. Receiver operating characteristic (ROC) curve analysis was conducted to assess the utility of age, Ct value, and D-dimer level in predicting encephalitis. Cox regression was used to identify independent factors associated with the risk of developing encephalitis, with consideration of multiple variables simultaneously. A *p*-value < 0.05 was considered to indicate statistical significance.

## 3. Results

A total of 450 critically ill patients with RT-qPCR-confirmed SARS-CoV-2 infection admitted to the ICU were analyzed. Among them, 96 patients were initially suspected of having encephalitis, and a definitive diagnosis based on both radiological and lumbar puncture findings was established in 38 cases. Therefore, encephalitis was present in 47.5% (38/96) of the suspected cohort. Positive cerebrospinal fluid findings indicated elevated protein levels; however, cell count, glucose levels, the immunoglobulin G index, and albumin levels remained within normal limits. PCR tests for SARS-CoV-2 and other infectious specimens were negative. In addition, cerebrospinal fluid immunoglobulin G levels were within normal reference limits and oligoclonal bands were negative. Positive MRI findings included signal intensity abnormalities affecting the subcortical and deep white matter on fluid-attenuated inversion recovery images.

The patients in the E+ group (*n* = 38; mean 69 ± 15 years) were significantly older than those in the E− group (*n* = 58; mean 61 ± 12 years) (*p* = 0.006; Table 1). At ICU admission, several key laboratory parameters significantly differed between groups. The median SARS-CoV-2 Ct value was significantly lower in the E+ group (23.7, IQR: 12.7–26.8) than the E− group (27.0, IQR: 23.8–31.5) (*p* < 0.001), thus indicating a higher viral load in the E+ group. Similarly, the median D-dimer levels were significantly higher in the E+ group (4.6, IQR: 2.4–11.1) than the E− group (1.1, IQR: 0.62–2.7) (*p* < 0.001).

Other baseline characteristics, including the sex distribution, body mass index (BMI), APACHE II score, SOFA score, and Charlson Comorbidity Index (CCI), did not show statistically significant differences between the E+ and E− groups. Most inflammatory markers and other laboratory values measured at ICU admission, such as the PaO_2_/FiO_2_ ratio, CRP, procalcitonin, leukocyte count, lymphocyte count, ferritin, and LDH, were also comparable between cohorts. Analysis of the cerebrospinal fluid in the E+ group revealed a median protein level of 64 mg/dL, indicating a mild-to-moderate elevation. In contrast, glucose levels and cell counts remained within normal limits.

Analysis of clinical outcomes indicated that patients in the E+ group experienced significantly longer durations of mechanical ventilation (median 19 days vs. 14 days; *p* = 0.006) and longer ICU stays (median 21 days vs. 15 days; *p* = 0.009) than the E− group. Although substantial overall mortality was observed in both groups, no statistically significant difference in mortality rates was found (50.0% [19/38 patients] in the E+ group versus 56.9% [33/58 patients] in the E− group; *p* = 0.507).

The predictive utility of age, Ct value, and D-dimer level for encephalitis was assessed with ROC curve analysis (Table 2). The area under the curve (AUC) was 0.65 (95% CI: 0.53–0.77; *p* = 0.02) for age ≥ 70 years, 0.71 (95% CI: 0.60–0.82; *p* = 0.005) for Ct ≤ 26.2, and 0.82 (95% CI: 0.72–0.92; *p* < 0.001) for D-dimer >2.3. Multivariate analysis further revealed that a Ct ≤ 26.2 (hazard ratio: 4.4, 95% CI: 1.2–16.3; *p* = 0.008) and D-dimer level >2.3 (hazard ratio: 5.8, 95% CI: 1.6–21.5; *p* < 0.001) at ICU admission were independently associated with a significantly elevated risk of developing encephalitis (Table 3). The reported cut-off values for D-dimer and Ct value were determined using Receiver Operating Characteristic (ROC) curve analysis to identify the point offering the optimal balance between sensitivity and specificity for predicting the development of COVID-19-associated encephalitis.

## 4. Discussion

This study aimed to investigate the relationship between viral load and the development of encephalitis in adult COVID-19 patients. Out of 450 patients analyzed, encephalitis was diagnosed in 38 cases, representing a substantial 8.44% of all COVID-19 patients admitted to the ICU, and 47.5% of those initially suspected. The high prevalence highlights the critical need for thorough neurological assessments in critically ill COVID-19 patients. Our findings indicate that patients who developed encephalitis were significantly older, presented with higher viral loads (lower Ct values), and showed signs of increased clotting activation with elevated D-dimer levels upon ICU admission.

SARS-CoV-2 viral load appeared to serve as a significant indicator for encephalitis among the laboratory parameters assessed at ICU admission, with lower median Ct values (*p* < 0.001) observed, indicating a higher viral load. The utility of Ct values as a prognostic indicator is subject to debate, with some studies presenting conflicting results. Normally, SARS-CoV-2 Ct values are not reported to the treating team, and a study suggests that reporting Ct values may help identify patients who need antiviral therapy [12]. While a direct causal link between viral load and encephalitis remains to be firmly established, respiratory tract viral load has been consistently reported as a determinant of illness severity in COVID-19 [10,13,14,15]. This pattern is also observed with other viral infections [16,17]. Furthermore, a reduction in viral load has been associated with improved clinical outcomes in acute respiratory tract infections [17]. In contrast, another study found no significant difference in viral load kinetics across varying COVID-19 severities [18]. Supporting this, a retrospective study of 202 hospitalized COVID-19 patients reported no significant association between initial SARS-CoV-2 Ct values and patients’ clinical presentation; their patient number was low, and the study design was different [19]. Between viral infections, viral load may be less or more relevant than other intrinsic patient factors in determining worse clinical outcomes [20]. Furthermore, viral load at a given time can be similar between asymptomatic and symptomatic cases [21]. The differences between studies might be related to the technique of sample collection, variation in the testing methods, variations in techniques and runs, and timing of the samples collected as reported previously [22]. Nevertheless, the dynamic interplay between viral load and host immune responses means that single-point measurements may be insufficient for a comprehensive understanding. In addition, the timing of the testing and calculation of the Ct values relative to symptoms would affect the level of the Ct values [23]. PCR values obtained at the time of inflammation severe enough to cause central nervous system involvement likely indicate a higher viral load. Host response over time offers a more robust approach to elucidating the complex interplay between virus and host [24,25].

The association between a higher viral burden and encephalitis may suggest direct viral neuroinvasion or a more robust viremic phase contributing to central nervous system involvement. There is evidence that SARS-CoV-2 can directly impact the brain, rather than exclusively through post-infectious inflammatory or autoimmune mechanisms [1,26,27]. While viral shedding can also be detected in other body fluids [28], confirming viral encephalitis in COVID-19 can be challenging due to the transient dissemination of SARS-CoV-2 and often low viral titers in cerebrospinal fluid [29]. SARS-CoV-2 can exert neurological manifestations through two primary mechanisms: The virus can directly enter the CNS, possibly via the olfactory bulb after nasal infection, facilitated by unknown factors. The olfactory bulb, uniquely unprotected by the dura mater, can become inflamed and demyelinated upon infection [30,31,32]. SARS-CoV-2-ACE2 receptor-mediated vascular damage following viremia may also contribute to involvement. This mechanism involves a significant immune response, with upregulated inflammatory mediators leading to a cytokine storm syndrome [33,34]. Moreover, an increasing pathogen load can independently drive the pathogenesis of an infection [35]. Even in the absence of direct neurotrophic effects, the sheer pathogen load is understood to be a critical trigger for the host inflammatory cascade. This implies a dose-dependent relationship where an elevated viral load correlates with amplified neuroinflammation. Therefore, understanding the determinants of pathogen load and its relationship with disease severity is crucial for comprehending these dynamics [7].

A notable finding was the significantly advanced age of patients with encephalitis (mean 69 years) compared to those without (mean 61 years) (*p* = 0.006). This observation is consistent with existing literature suggesting that older age is a significant risk factor for severe COVID-19 outcomes and neurological complications [36,37]. Furthermore, there is evidence of longer duration of viral shedding in more severe cases and older individuals [10,38]. The precise mechanisms linking age to increased susceptibility to COVID-19 encephalitis warrant further investigation. Potential contributing factors include age-related immune dysregulation, compromised integrity of the blood–brain barrier, or a higher prevalence of comorbidities in older individuals. Interestingly, a separate investigation into African Swine Fever (ASF) found that despite an age-related trend in viral replication, there was no corresponding correlation between viral load and the extent of pathological lesions across multiple organs [39]. This suggests that the relationship between viral load and organ damage might differ depending on the specific viral pathogen.

ICU severity scores (APACHE II, SOFA) and inflammatory markers (CRP, procalcitonin, leukocyte/lymphocyte counts, ferritin, LDH) did not significantly differ between the groups. This suggests that while overall systemic inflammation and organ dysfunction are characteristic of critical COVID-19, their general magnitude may not specifically differentiate patients who develop encephalitis from those who do not. Instead, the specific patterns of viral burden and coagulopathy, as indicated by Ct and D-dimer, appear to be more distinctive. The heightened D-dimer (*p* < 0.001) in encephalitis patients may reflect increased inflammatory processes in COVID-19 patients, microthrombosis within the cerebral vasculature, or widespread endothelial damage that contributes to neuroinflammation and blood–brain barrier disruption [40,41].

Regarding clinical outcomes, patients with encephalitis experienced significantly prolonged durations of mechanical ventilation and ICU duration. This underscores the substantial burden that encephalitis imposes on critical care resources and highlights the increased morbidity associated with this neurological complication. Although the mortality rate was high in both groups (around 50–57%), there was no statistically significant difference, suggesting that while encephalitis prolongs illness and resource utilization, it may not independently drive a higher mortality rate beyond the severe critical illness already present in this ICU cohort. It is plausible that the high baseline mortality in critically ill COVID-19 patients may mask the individual impact of encephalitis on survival in this specific cohort size. Severity of respiratory impairment and neuronal damage are the factors that seem to determine the worst prognosis [1].

The predictive utility analysis further strengthened the role of Ct and D-dimer. D-dimer exhibited the highest discriminatory power (AUC = 0.82), followed by Ct (AUC = 0.71), and age (AUC = 0.65). The Cox proportional hazards model demonstrated that a lower Ct value and elevated D-dimer levels were independently associated with a significantly increased risk of developing encephalitis (Hazard Ratios of 1.9 and 2.9, respectively). While both markers are useful, the high Negative Predictive Value (NPV) of D-dimer suggests it is a particularly valuable tool for identifying patients at low risk for neurological complications.

The interplay between respiratory failure and central nervous system complications in COVID-19 mirrors the pathophysiology observed in other chronic and acute respiratory conditions. As noted by Corlateanu et al. (2018) [42] in the context of COPD and stroke, systemic inflammation originating in the lungs creates a ‘pro-thrombotic milieu’ that may predispose the cerebral vasculature to injury. In our cohort, the combination of a high SARS-CoV-2 viral load and elevated D-dimer may suggest a similar mechanism. Furthermore, while our study focused on severe encephalopathy, the neuroinflammatory pathways triggered by high viral loads may also explain more common, subacute symptoms such as dizziness and fatigue. If high viral replication at admission leads to early, low-grade neuroinflammation or altered cerebral perfusion, it could account for the pervasive ‘brain fog’ and vestibular symptoms reported even in patients who do not progress to full encephalitis/encephalopathy.

### Limitation

A primary limitation of this study is the selective approach and timing of neurological evaluation. Due to the critically ill nature of the cohort, definitive investigations (MRI and LP) were intentionally delayed and performed only in patients whose altered mental status persisted beyond 48 h after sedation discontinuation or whose delirium was refractory to management. This necessary selection bias means that our study focuses exclusively on the most severe or persistent neurological complications and likely underestimates the true overall prevalence of COVID-19-associated CNS involvement. The reported incidence (e.g., 8.44%) thus represents the burden of subacute/delayed and clinically severe encephalopathy requiring specialized ICU investigation, rather than the total incidence across all stages of illness severity. Another limitation concerns the single-point nasopharyngeal Ct value used as a proxy for viral burden. Ct values provide a semi-quantitative estimate of viral RNA presence; they are influenced by sampling technique and the timing of the illness. The single measurement at ICU admission precludes the assessment of viral kinetics, critical predictors of host response. The retrospective study design prevented the collection of longitudinal viral load data. We also did not specifically examine autoimmune markers, which might have roles in encephalitis. Lastly, we could not reliably correlate vaccination status with neurological outcomes.

## 5. Conclusions

Our findings suggested that lower Ct values, indicating higher viral loads, were significantly associated with elevated risk of encephalitis. Additionally, older age and elevated D-dimer levels were associated with further increases in encephalitis risk. These findings highlight the importance of considering viral load, age, and D-dimer levels in assessing the risk of neurological complications in patients with COVID-19. Enhanced awareness might improve patient outcomes by facilitating more effective monitoring and treatment. Further prospective studies are warranted to elucidate the mechanisms through which high viral load exacerbates encephalitis.

## Figures and Tables

**Table 1:** Comparative analysis of demographic, clinical, and laboratory characteristics between patient groups.

	Encephalitis (−) (*n* = 58)	Encephalitis (+) (*n* = 38)	*p*
Age, years	61 ± 12	69 ± 15	0.006
Male, *n* (%)	42 (72.4)	26 (68.4)	0.674
BMI, kg/m^2^	26.4 (24.7–28.7)	26.0 (24.4–27.9)	0.412
APACHE II	17 ± 6	19 ± 3	0.098
SOFA Score	7 (6–8)	7 (7–8)	0.326
CCI	4 (2–6)	5 (3–7)	0.113
At the ICU admission			
Ct	27.0 (23.8–31.5)	23.7 (12.7–26.8)	<0.001
PaO_2_/FiO_2_ ratio	130 (107–177)	146 (110–180)	0.102
CRP	13.6 (7.1–19.1)	10.7 (5.4–19.1)	0.212
Procalcitonin	0.20 (0.08–0.52)	0.28 (0.09–0.95)	0.328
Leucocyte count	10.4 (7.3–14.1)	12.2 (8.1–13.9)	0.391
Lymphocyte count	0.54 (0.39–0.73)	0.59 (0.46–0.85)	0.225
Ferritin	903 (660–1363)	986 (308–4381)	0.846
D-dimer	1.1 (0.62–2.7)	4.6 (2.4–11.1)	<0.001
LDH	459 (351–593)	341 (241–711)	0.238
Outcomes			
Duration of MV	14 (9–19)	19 (14–27)	0.006
Length of ICU stay	15 (13–21)	21 (16–28)	0.009
Mortality, *n* (%)	33 (56.9)	19 (50.0)	0.507

MV: Mechanical ventilation.

**Table 2:** ROC curve analysis indicating the ability of the variables age, Ct, and D-dimer to predict a certain outcome.

	Cut-Off Values	AUC (95% CI)	Sensitivity %	Specificity %	PPV %	NPV %	*p*
Age, years	>70	0.67 (0.56–0.79)	62	68	55	74	0.004
Ct	≤26.2	0.71 (0.60–0.82)	74	64	58	79	0.001
D-dimer	>2.3	0.78 (0.68–0.88)	81	72	65	86	<0.001

AUC: Area Under the Curve; Ct: Cycle Threshold.

**Table 3:** Cox proportional hazards model.

	Hazard Ratio (95% CI)	*p*
Age > 70	1.7 (0.9–3.5)	0.117
Ct ≤ 26.2	1.9 (1.1–4.4)	0.032
D-dimer > 2.3	2.9 (1.3–6.6)	0.010

## Data Availability

The data supporting the findings of this study are available from the corresponding author upon reasonable request and with the permission of the local ethics committee.
